# Seasonal COVID-19 surge related hospital volumes and case fatality rates

**DOI:** 10.1186/s12879-022-07139-2

**Published:** 2022-02-23

**Authors:** Joseph E. Ebinger, Roy Lan, Matthew Driver, Nancy Sun, Patrick Botting, Eunice Park, Tod Davis, Margo B. Minissian, Bernice Coleman, Richard Riggs, Pamela Roberts, Susan Cheng

**Affiliations:** 1 Department of Cardiology, Cedars-Sinai Medical Center, Los Angeles, CA USA; 2 Smidt Heart Institute, Cedars-Sinai Medical Center, Los Angeles, CA USA; 3 College of Medicine, University of Tennessee Health Science Center, Memphis, TN USA; 4 Enterprise Data Intelligence, Cedars-Sinai Medical Center, Los Angeles, California, USA; 5 Brawerman Nursing Institute and Nursing Research Department, Cedars-Sinai Medical Center, Los Angeles, CA USA; 6 Department of Medical Affairs, Cedars-Sinai Medical Center, Los Angeles, CA USA; 7 Department of Biomedical Sciences, Division of Informatics, Cedars-Sinai Medical Center, Los Angeles, CA USA

**Keywords:** COVID-19, Surge, Case fatality

## Abstract

**Background:**

Seasonal and regional surges in COVID-19 have imposed substantial strain on healthcare systems. Whereas sharp inclines in hospital volume were accompanied by overt increases in case fatality rates during the very early phases of the pandemic, the relative impact during later phases of the pandemic are less clear. We sought to characterize how the 2020 winter surge in COVID-19 volumes impacted case fatality in an adequately-resourced health system.

**Methods:**

We performed a retrospective cohort study of all adult diagnosed with COVID-19 in a large academic healthcare system between August 25, 2020 to May 8, 2021, using multivariable logistic regression to examine case fatality rates across 3 sequential time periods around the 2020 winter surge: pre-surge, surge, and post-surge. Subgroup analyses of patients admitted to the hospital and those receiving ICU-level care were also performed. Additionally, we used multivariable logistic regression to examine risk factors for mortality during the surge period.

**Results:**

We studied 7388 patients (aged 52.8 ± 19.6 years, 48% male) who received outpatient or inpatient care for COVID-19 during the study period. Patients treated during surge (N = 6372) compared to the pre-surge (N = 536) period had 2.64 greater odds (95% CI 1.46–5.27) of mortality after adjusting for sociodemographic and clinical factors. Adjusted mortality risk returned to pre-surge levels during the post-surge period. Notably, first-encounter patient-level measures of illness severity appeared higher during surge compared to non-surge periods.

**Conclusions:**

We observed excess mortality risk during a recent winter COVID-19 surge that was not explained by conventional risk factors or easily measurable variables, although recovered rapidly in the setting of targeted facility resources. These findings point to how complex interrelations of population- and patient-level pandemic factors can profoundly augment health system strain and drive dynamic, if short-lived, changes in outcomes.

**Supplementary Information:**

The online version contains supplementary material available at 10.1186/s12879-022-07139-2.

## Background

Adverse clinical outcomes, particularly case fatality, are known to increase during periods of strain on healthcare systems caused by excess patient volume [1, 2]. The COVID-19 pandemic has led to especially profound challenges, many related to the uniquely evolving features of SARS-CoV-2 infection, with numerous hospitals having experienced substantially greater COVID-19 case fatality during periods of regional surges. However, the vast majority of published reports on the relationship between hospital volume and excess mortality risk have been focused almost exclusively on data collected during the initial months of the pandemic—prior to the implementation of more developed standards of care [3–6]. The earlier reports also tended to highlight data from facilities with limited staff and operational resources; these factors are likely to have contributed to greater increases in mortality during periods of surge [7], leading to potentially extreme estimates of excess mortality associated with rapid increases in patient volume for a given health system [8, 9]. Reports from the initial phase of the pandemic are also limited to the effects of the earlier SARS-CoV-2 variants, and more recently emerged variants are known to have differential impacts on clinical outcomes [10].

Amidst ongoing regional surges of COVID-19, related in part to recently emerged SARS-CoV-2 variants, more uptodate information is needed regarding how the pressures of COVID-19 surges on health systems can impact outcomes—especially during the winter season, when colder weather tends to increase both viral transmissibility and patient-level susceptibility to more severe types of illness [11]. Most hospitals have adopted more advanced SARS-CoV-2 therapies, developed standards of care for more severely ill patients, and developed protocols for anticipating rapid increases in patient volume. However, the more transmissible SARS-CoV-2 variants and the overall epidemiologic persistence of COVID-19 across all communities have led to surges that continue to impose dynamic challenges for all health systems.

## Methods

### Study design and sampling

To investigate the nature and correlates of COVID-19 associated outcomes before, during, and after the Winter 2020 surge, we performed a retrospective cohort study of all adult patients (age ≥ 18 years) treated for confirmed COVID-19 infection in our large multisite healthcare system based in Los Angeles, California (Cedars-Sinai Health System), from August 25, 2020 through May 8, 2021. Cedars-Sinai Medical Center is the largest non-profit hospital in the western United States, with a total of 886 hospital beds, 96 of which are in intensive care units (ICU). In addition, it has a catchment area of > 1.8 million people, with over a quarter million inpatient hospital days for admitted patients, > 90,000 emergency department visits, and nearly 800,000 outpatient appointments annually. All laboratory testing for COVID-19 were performed using reverse transcriptase polymerase chain reaction (PCR) of extracted RNA from nasopharyngeal swabs.

### Data collection

We obtained demographic, clinical, and outcomes data from the Cedars-Sinai electronic health record (EHR) and manually confirmed key clinical and outcomes variables. We defined race/ethnicity membership as follows: Asian, Hispanic/Latinx ethnicity (all races), non-Hispanic Black, non-Hispanic White, and other (including individuals with multiple races listed). To estimate relative comorbid status, the Elixhauser Comorbidity Index score was calculated with van Walraven weighting, using the International Classification of Diseases-10 (ICD-10) codes present at the time of COVID-19 presentation [12]. Specific clinical characteristics were identified for each patient using ICD-10 diagnoses at presentation, including: obesity, hypertension, diabetes mellitus, prior myocardial infarction (MI) or heart failure (HF), and prior chronic obstructive pulmonary disease (COPD) or asthma. Laboratory values from the time of admission were also obtained from the EHR.

### Exposures and outcomes

Our primary exposure was the receipt of care for COVID-19 during three distinct time periods: pre-surge (August 25, 2020–November 7, 2020), surge (November 8, 2020–February 22, 2021), and post-surge (February 23, 2021–May 8, 2021). The start of the surge period was declared by hospital capacity management based on trends in internal and regional case volumes. The end of the surge period was calculated as the date at which the 7-day rolling average of newly diagnosed COVID-19 cases dropped below the 7-day rolling average at the beginning of the surge. The pre-surge period and post-surge period were defined as the 75 days before and after the surge period, respectively, as a larger observation window would include cases from a prior surges.

Our primary outcome was COVID-19 case fatality. For patients admitted to the hospital, we defined case fatality as a death during hospitalization or up to 30 days from the time of discharge as documented in the EHR. For patients not requiring admission, case fatality was defined as death within 30 days from the date of initial COVID-19 diagnosis.

### Statistical analyses

Demographic and clinical characteristics were summarized using mean and standard deviation (SD) for continuous variables and as counts with percentages for all categorical variables. We compared demographic, clinical and laboratory characteristics across time periods using analysis of variance (ANOVA) for continuous measures and Chi-squared tests for categorical measures. We conducted multivariable logistic regression to examine the association between time period and case fatality, both overall and by subgroups of patients admitted to the hospital and those receiving ICU-level care. Additionally, we used multivariable logistic regression to examine risk factors for mortality during the surge period. All analyses were adjusted for age, sex, race/ethnicity, Elixhauser score, hypertension, and diabetes mellitus. A two-tailed P-value of < 0.05 were considered significant. All analyses were conducted using R V.4.0.2 (R Foundation for Statistical Computing, Vienna, Austria).

## Results

### Cohort characteristics

**Fig. 1:**
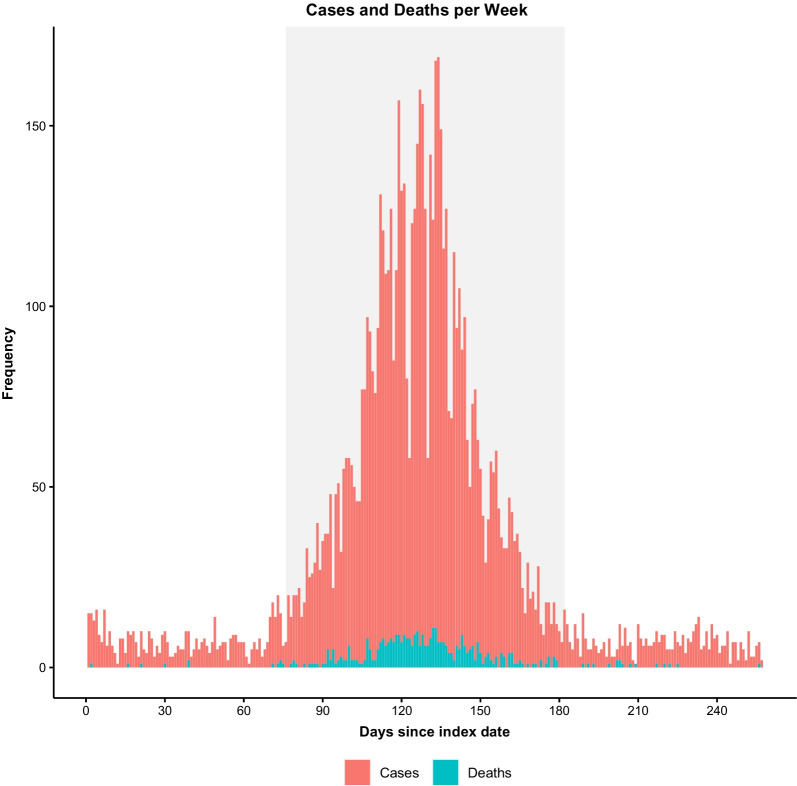
COVID-19 cases and deaths per week

A total of 7,388 patients with COVID-19 were identified during the study period with a mean age of 52.8 ± 19.6 years, 47.8% of whom were male. A total of 536 patients were diagnosed during the pre-surge period, 6372 during the surge, and 480 during the post-surge period (Fig. 1). Overall, patients in the surge period were on average older (53.3 ± 19.5) than those in the pre-surge (50.3 ± 19.8) and post-surge periods (48.5 ± 19.5; p < 0.001). Patients during the surge period also exhibited greater rates of obesity, hypertension and diabetes mellitus when compared to the pre-surge and post-surge periods. The average mean daily COVID-19 case count increased (p < 0.001) during the surge period (59.6 ± 41.6), compared to both the pre-surge period (7.1 ± 3.7) and the post-surge period (6.4 ± 3.1) (Table 1). Transfers from other acute care hospitals ranged between 2.5% and 4.9% of all COVID-19 cases across the 3 periods (Additional file 1: Table S1).

**Table 1:** Demographic and clinical characteristics of COVID-19 patients, 8/25/2020 to 5/8/2021

	Overall (n = 7388)	Pre-surge period (n = 536)	Surge period (n = 6372)	Post-surge period (n = 480)	p-value
Average daily cases, mean (SD)	28.7 (37.5)	7.1 ( 3.7)	59.6 (41.6)	6.4 ( 3.1)	< 0.001
Demographic characteristics					
Age, mean (SD), years	52.76 (19.60)	50.26 (19.78)	53.29 (19.53)	48.48 (19.52)	< 0.001
Male sex, n (%)	3529 (47.8)	260 (48.5)	3054 (47.9)	215 (44.8)	0.389
Race/ethnicity, n (%)					
Asian	624 (8.4)	31 (5.8)	564 (8.9)	29 (6.0)	< 0.001
Hispanic/Latinx	2423 (32.8)	158 (29.5)	2136 (33.5)	129 (26.9)	
Non-Hispanic Black	1212 (16.4)	97 (18.1)	1034 (16.2)	81 (16.9)	
Non-Hispanic White	2416 (32.7)	193 (36.0)	2031 (31.9)	192 (40.0)	
Other	368 (5.0)	21 (3.9)	324 (5.1)	23 (4.8)	
Clinical characteristics					
Elixhauser comorbidity score^a^, mean ± SD	7.44 (11.84)	6.31 (10.24)	7.50 (11.95)	7.82 (11.98)	0.060
Obesity, n (%)	1697 (23.0)	109 (20.3)	1498 (23.5)	90 (18.8)	0.019
Hypertension, n (%)	3001 (40.6)	176 (32.8)	2648 (41.6)	177 (36.9)	< 0.001
Diabetes mellitus, n (%)	1804 (24.4)	105 (19.6)	1597 (25.1)	102 (21.2)	0.004
Prior myocardial infarction or heart failure, n (%)	1230 (16.6)	70 (13.1)	1073 (16.8)	87 (18.1)	0.052
Prior COPD or asthma, n (%)	1326 (17.9)	78 (14.6)	1174 (18.4)	74 (15.4)	0.026

COPD, chronic obstructive pulmonary disease; SD, standard deviation

^a^Elixhauser comorbidity score calculated using the van Walraven method

### Multivariable analysis

During the study period there were 412 deaths (case fatality rate 5.6%), with 11 (2.1%) during the pre-surge period, 385 (6.0%) during the surge period, and 16 (3.3%) during the post-surge period. Following multivariable adjustment for demographic and clinical characteristics, patients diagnosed with COVID-19 during the surge period experienced higher odds of death (OR: 2.64, 95% CI 1.46–5.27) compared to patients diagnosed in the pre-surge time period (Table 2). Odds of death were also higher during the surge period for hospitalized patients (3.20, 1.76–6.43), and those admitted to the ICU, (2.81, 1.20–7.29) (Additional file 1: Table S2). No statistically significant differences in case fatality were observed between the pre-surge and post-surge periods in the overall, hospitalized, and ICU groups.

**Table 2:** Odds of death, by time period, among patients with COVID-19

	Unadjusted OR (95% CI)	Adjusted OR (95% CI)^a^
Time period		
Pre-surge (8/25/2020–11/7/2020)	Ref.	Ref.
Surge (11/8/2020–2/22/2021)	3.07 (1.76, 5.98)	**2.64 (1.46, 5.27)**
Post-surge (2/23/2021–5/8/2021)	1.65 (0.76, 3.68)	1.63 (0.72, 3.81)

Bold value indicate odds ratios who’s 95% CI does not cross unity, indicating statistical significance

CI, Confidence Interval; OR, Odds Ratio

^a^Model adjusted for age, sex, race/ethnicity, Elixhauser score, hypertension, diabetes, obesity, chronic obstructive pulmonary disease or asthma, and prior myocardial infarction or heart failure

In the fully adjusted model, during the surge period patients over age 65 (5.76, 4.29–7.81), males (1.55, 1.23–1.96), Hispanic/Latinx patients (1.64, 1.05–2.50), and Asian patients (1.64, 1.05–2.50) were more likely to experience death. Increasing comorbidity burden, as assessed by Elixhauser score, was also positively associated with risk of death (1.06, 1.05–1.07) (Table 3).

**Table 3:** Risk factors for death during surge among patients with COVID-19

	Unadjusted OR (95% CI)	Adjusted OR (95% CI)^a^
Age		
Below 65	Ref.	Ref.
Above 65	10.91 (8.47, 14.22)	**5.76 (4.29, 7.81)**
Sex		
Female	Ref.	Ref.
Male	1.84 (1.49, 2.28)	**1.55 (1.23, 1.96)**
Race/ethnicity		
Non-Hispanic white	Ref.	Ref.
Non-Hispanic Black	0.57 (0.41, 0.79)	0.72 (0.50, 1.02)
Hispanic/Latinx	0.64 (0.50, 0.82)	**1.53 (1.15, 2.05)**
Asian	0.74 (0.50, 1.07)	**1.64 (1.05, 2.50)**
Other	0.76 (0.46, 1.20)	1.17 (0.67, 1.96)
Elixhauser comorbidity score	1.08 (1.08, 1.09)	**1.06 (1.05, 1.07)**
Diabetes		
No	Ref.	Ref.
Yes	3.55 (2.88, 4.38)	1.19 (0.93, 1.53)
Hypertension		
No	Ref.	Ref.
Yes	4.25 (3.38, 5.38)	0.96 (0.72, 1.29)

Bold values indicate odds ratios who’s 95% CI does not cross unity, indicating statistical significance

CI, Confidence Interval; OR, Odds Ratio

^a^Model adjusted for age, sex, race/ethnicity, Elixhauser score, hypertension, diabetes, obesity, chronic obstructive pulmonary disease or asthma, and prior myocardial infarction or heart failure

### First-encounter measures of illness severity

A total of 2537 patients were hospitalized during the study period. To assess the severity of illness at the time of initial clinical presentation (i.e. first encounter), we examined the presenting vital signs and laboratory values at the time of hospital admission. Clinically modest but statistically significant differences were appreciated among patients across time periods, including for the average mean C-Reactive Protein (in mg/L 110.4 ± 85.4 surge, 84.8 ± 79.1 pre-surge, 101.0 ± 100.3 post-surge; p = 0.019), serum Creatinine (in mg/dL 1.6 ± 2.4, ± 1.2 ± 1.4, ± 1.7 ± 2.6, p = 0.031), serum HCO3 (in mmol/L 22.8 ± 5.7, 25.2 ± 6.0, 22.9 ± 8.7; p = 0.046), mean systolic blood pressure (in mmHg 124.8 ± 19.7, 123.1 ± 18.9, ± 121.3 ± 20.6, p = 0.039), mean respiratory rate (20.2 ± 4.1, 19.3 ± 3.7, 18.7 ± 3.4; p < 0.001), SPO2 (95.3 ± 3.4, 96.0 ± 2.6, 96.2 ± 3.9; p < 0.001), and mean temperature (degrees Fahrenheit 99.6 ± 1.4, 99.8 ± 1.5, ± 99.2 ± 1.4; p < 0.001) (Additional file 1: Table S3).

## Discussion

In this study of over 7000 patients who received outpatient or inpatient care for COVID-19 between August 2020 and May 2021, adjusted mortality risk increased significantly from the Fall pre-surge period to the Winter surge period—corresponding with the very rapid rise in patient volume. Adjusted mortality risk then returned to pre-surge levels during the Spring post-surge period—corresponding to a subsequent similarly rapid decline in patient volume. The excess mortality risk during the winter COVID-19 surge was not adequately explained by conventional sociodemographic or pre-existing risk traits or easily measurable variables. However, we did observe that the excess risk recovered rapidly in the setting of targeted facility resources. Notably, however, first-encounter patient-level measures of illness severity appeared higher during surge compared to non-surge periods—suggesting that timing of patient presentation, as related to timing illness onset, may have contributed along with external socioeconomic or other epidemiological factors to augmenting risk for adverse outcomes during the surge period.

Our findings extend from numerous earlier scientific and lay reports that have chronicled the overwhelming nature of the initial COVID-19 surge that began the United States in March of 2020 [13–16]. This first wave was compounded by multiple factors including lack of knowledge around appropriate treatment of SARS-CoV-2 infection, unprepared resource supply chains, and a lack of adequately trained personnel in highly impacted communities. Advances in standards of care including the use of monoclonal antibodies, steroids and Remdesivir [17], among others, as well as more robust supply chains [18, 19] were present during the Winter surge period evaluated in the current study—allowing for a more focused evaluation of the excess patient volume effect on COVID-19 case outcomes at a health system level. Further, the presence of ‘valleys’ in patient volume during non-surge time periods allowed for comparison of surge case fatality rates to those when excess patient volume was not a predominant factor.

Expanding longitudinally from the earlier reports, our analysis from the Winter 2020 surge found that patients treated for COVID-19 during the surge period had higher odds of death, both overall and when stratified by maximum level of care required (outpatient, inpatient, and ICU). Importantly, we observed that patients presenting for care during the surge were more likely to be older, male, Hispanic/Latinx, and with a greater burden of comorbidities than those presenting during the non-surge periods; all these factors have been linked to greater severity of COVID-19 illness [4, 20–22]. Nonetheless, odds of death remained elevated during the surge period even when adjusting for these risk factors. We also observed apparently modest but statistically significant differences in laboratory characteristics among patients hospitalized during the surge. It is well described that during periods of high COVID-19 activity in the community, patients delay seeking care due to fear of becoming ill or spreading the virus themselves [23–27]. As such, delayed presentations, particularly among vulnerable patient populations, with subsequent late initiation of COVID-19 specific therapies, may well have contributed to at least a portion of the observed excess mortality risk.

Previous studies have also found that discernible non-patient factors contribute to measurable variation in COVID-19 outcomes. Increased COVID-19 case rates [3, 28, 29], ICU strain [5, 12], and limited hospital resource availability, including number of hospital beds and staff [7], have been linked to increased case fatality, though these studies examine outcomes solely during the initial stages of the pandemic. Nonetheless, these phenomena are known to continue to impact COVID-19 outcomes across in at-risk regions and communities. Fortunately, although surges in COVID-19 patient volume required the transformation of previously non-critical care environments into advanced care locations within our health system, we were ultimately able to house and medically accommodate all patients requiring advanced care including intubation, mechanical ventilation, and mechanical circulatory support. These advanced care needs were met through redirecting staffing support from non-critical care to critical care settings. Beyond facility-level factors, lack of statistically significant differences in case fatality between pre- and post-surge periods suggest that the increase in case fatality was not related to secular trends in COVID-19 outcomes during the study period, such as improvements in the standard of care and the circulation of regional COVID-19 variants linked to increased mortality [9, 10]. In fact, while not statistically significant, we observed a trend towards slightly higher mortality during the post-surge compared to pre-surge period. This finding could have been related to a bias towards more severely ill patients presenting for medical encounters over time, or a residual excess in hospitalized patients; further studies using more detailed data are needed to clarify the factors contributing to variations in post-surge recovery periods.

Several limitations of this study merit consideration. Our data were derived from a single healthcare system, and thus our findings may not be generalizable to other populations, especially those outside the United States. However, our patient cohort was found to be diverse, both demographically as well as clinically, and our institution is a high-volume center serving a large and diverse urban population. Reliance on EHR data to identify deaths may result in misclassification, particularly by undercounting deaths occurring outside of the hospital, though we would expect this to attenuate rather than confound our results. We were unable to systematically capture data on timing of illness onset, which precluding assessment of symptom duration prior to presentation to medical care. We recognize that all the factors driving as well as correlated with delayed patient presentations (i.e. delays in patients seeking or receiving medical attention), especially during COVID-19 surge periods, are critically important to identify and yet not easily measured in the real-world community setting. Detailed data on temporal trends in hospital occupancy, medical care staffing (e.g. nurse-to-patient ratios), and medical care supplies and other resources were not available for the current analysis and will be important for future investigations of excess mortality during surge periods. Finally, we were unable to control for COVID-19 vaccination status as vaccines were not available for the majority of the cohort until the post-surge period, and vaccine uptake in the post-surge period may have lowered risk for severe outcomes. However, given that all patients in our cohort were COVID-positive and that reported breakthrough infection rates are relatively low [30], it is unlikely that enough patients in the post-surge period were vaccinated to have biased our results.

## Conclusions

In summary, our study highlights the reality of excess mortality risk seen during the last Winter surge of COVID-19 experienced by a high-volume healthcare system serving a diverse and large metropolitan region. The excess mortality risk was not explained by conventional risk factors or easily measurable variables, although recovered rapidly in the setting of targeted facility resources. These findings point to how complex interrelations of population- and patient-level pandemic factors can profoundly augment health system strain and drive dynamic, if short-lived, changes in outcomes.

## Supplementary Information

**Additional file 1: Table S1.** COVID-19 patients transferred from other acute care facilities and associated mortality, by time period. **Table S2.** Odds of death, by time period, among patients with COVID-19 among those admitted to the hospital and to those admitted to the ICU. **Table S3.** Diagnostic values at admission among patients hospitalized for COVID-19, by time period.

## Data Availability

Due to their sensitive nature, restrictions apply to the availability of the data that support the findings, which were used under license for the current study, and so are not publicly available. Data are however available from the authors upon reasonable request and with permission of Cedars-Sinai Medical Center.

## Abbreviations

ANOVA: Analysis of variance
COPD: Chronic obstructive pulmonary disease
EHR: Electronic health record
ICD-10: International Classification of Diseases-10
ICU: Intensive care units
HF: Heart failure
MI: Myocardial infarction
PCR: Polymerase chain reaction
SD: Standard deviation
