# Supplementary material for: Seasonal COVID-19 surge related hospital volumes and case fatality rates
Source: BMC Infect Dis. 2022 Feb 23;22:178. doi: 10.1186/s12879-022-07139-2 (PMC8864601; doi:10.1186/s12879-022-07139-2)
Supplement: Supplementary file 1 — Additional file 1: Table S1. COVID-19 patients transferred from other acute care facilities and associated mortality, by time period. Table S2. Odds of death, by time period, among patients with COVID-19 among those admitted to the hospital and to those admitted to the ICU. Table S3. Diagnostic values at admission among patients hospitalized for COVID-19, by time period. [file 12879_2022_7139_MOESM1_ESM.docx]

**Seasonal COVID-19 Surge Related Hospital Volumes and Case Fatality Rates**

**Correspondence:** Joseph E. Ebinger, Department of Cardiology, Smidt Heart Institute, Cedars Sinai Medical Center, Los Angeles, CA, USA, Phone: 310-423-2726**,** Email: joseph.ebinger@csmc.edu

**Table S1.** COVID-19 patients transferred from other acute care facilities and associated mortality, by time period.

|  | **Overall  (n = 2537)** | **Pre-surge period  (n = 203)** | **Surge period  (n = 2149)** | **Post-surge period  (n = 185)** |
| --- | --- | --- | --- | --- |
|  |  |  |  |  |
| Transfers, n (%) as percent of admitted patients | 71 (2.8) | 10 (4.9) | 53 (2.5) | 8 (4.3) |
|  |  |  |  |  |
| Mortality rate, n (%), all hospitalized patients | 394 (15.5) | 11 (5.4) | 368 (17.1) | 15 (8.1) |
|  |  |  |  |  |
| Transfer mortality rate, n (%) | 17 (23.9) | 2 (20.0) | 12 (22.6) | 3 (37.5) |
|  |  |  |  |  |
| Proportional mortality of transfer patients, n (%) as percent of all deaths among hospitalized patients | 17 (4.3) | 2 (18.2) | 12 (3.3) | 3 (20.0) |

**Table S2.** Odds of death, by time period, among patients with COVID-19 among those admitted to the hospital and to those admitted to the ICU.

|  | **Unadjusted OR (95% CI)** | **Adjusted OR (95% CI)*** |
| --- | --- | --- |
| **All Hospitalized Patients** |  |  |
| Pre-surge (8/25/2020 - 11/7/2020) [n = 203] | Ref. | Ref. |
| Surge (11/8/2020 - 2/22/2021) [n = 2149] | 3.61 (2.04, 7.10) | **3.20 (1.76, 6.43)** |
| Post-surge (2/23/2021 - 5/8/2021) [n = 185] | 1.54 (0.69, 3.53) | 1.69 (0.73, 4.02) |
|  |  |  |
| **Patients admitted to the ICU** |  |  |
| Pre-surge (8/25/2020 - 11/7/2020) [n = 38] | Ref. | Ref. |
| Surge (11/8/2020 - 2/22/2021) [n = 437] | 3.31 (1.56, 7.90) | **2.81 (1.20, 7.29)** |
| Post-surge (2/23/2021 - 5/8/2021) [n = 40] | 1.42 (0.50, 4.16) | 1.21 (0.38, 3.95) |

CI (Confidence Interval); OR (Odds Ratio)

*Model adjusted for age, sex, race/ethnicity, Elixhauser score, hypertension, diabetes, obesity, chronic obstructive pulmonary disease or asthma, and prior myocardial infarction or heart failure.

**Table S3.** Diagnostic values at admission among patients hospitalized for COVID-19, by time period

| **Lab value*** | **Overall  (n = 2537)** | **Pre-surge period  (n = 203)** | **Surge period  (n = 2149)** | **Post-surge period  (n = 185)** | **p-value** |
| --- | --- | --- | --- | --- | --- |
|  |  |  |  |  |  |
| B-type natriuertic peptide, mean (SD), pg/mL | 332.68 (1024.81) | 193.50 (577.30) | 341.91 (1063.04) | 388.61 (865.62) | 0.231 |
| C-reactive protein, mean (SD), mg/L | 107.49 (85.75) | 84.80 (79.05) | 110.41 (85.36) | 101.01 (100.30) | 0.019 |
| Creatinine, mean (SD), mg/dL | 1.59 (2.32) | 1.19 (1.44) | 1.62 (2.35) | 1.74 (2.65) | 0.031 |
| D-dimer, mean (SD), ug/mL FEU | 2.21 (2.97) | 2.13 (4.62) | 2.23 (2.89) | 2.11 (1.70) | 0.982 |
| Interleukin-6, mean (SD), pg/mL | 50.57 (162.98) | 50.80 (105.55) | 50.13 (180.99) | 56.09 (79.65) | 0.992 |
| ESR, mean (SD), mm/hr | 53.80 (33.44) | 39.69 (26.54) | 54.42 (31.96) | 52.72 (51.53) | 0.294 |
| Ferritin, mean (SD), ng/mL | 1439.06 (4342.19) | 799.24 (999.46) | 1553.74 (4712.41) | 972.61 (1476.53) | 0.185 |
| HCO3, mean (SD), mmol/L | 22.99 (5.89) | 25.15 (6.02) | 22.81 (5.67) | 22.90 (8.65) | 0.046 |
| Hemoglobin, mean (SD), % of total | 8.04 (2.66) | 7.10 (2.20) | 8.17 (2.69) | 7.48 (2.61) | 0.089 |
| Lactate dehydrogenase, mean (SD), IU/L | 413.22 (431.13) | 366.72 (200.12) | 427.18 (472.45) | 337.11 (167.84) | 0.249 |
| Procalcitonin, mean (SD), ng/mL | 1.29 (9.05) | 0.43 (1.98) | 1.35 (9.56) | 1.58 (4.66) | 0.560 |
| Sodium, mean (SD), mmol/L | 136.72 (5.41) | 136.16 (4.52) | 136.79 (5.55) | 136.45 (4.53) | 0.240 |
| Total CO2, mean (SD), mEq/L | 23.60 (4.49) | 24.23 (4.36) | 23.58 (4.48) | 23.13 (4.74) | 0.057 |
| White blood cell count, mean (SD), thousand per uL | 8.59 (7.54) | 7.46 (4.06) | 8.67 (7.98) | 8.86 (4.53) | 0.082 |
| Pulse rate, mean (SD), beats per minute | 81.47 (16.23) | 80.75 (16.26) | 81.63 (16.36) | 80.31 (14.53) | 0.458 |
| Systolic blood pressure, mean (SD), mmHg | 124.42 (19.74) | 123.07 (18.86) | 124.82 (19.73) | 121.28 (20.55) | 0.039 |
| Diastolic blood pressure, mean (SD), mmHg | 71.59 (12.78) | 70.36 (12.53) | 71.80 (12.77) | 70.53 (13.08) | 0.155 |
| Respiratory rate, mean (SD), breaths per minute | 20.03 (4.01) | 19.30 (3.67) | 20.21 (4.06) | 18.73 (3.41) | <0.001 |
| SPO2, mean (SD), percent | 95.40 (3.37) | 96.04 (2.60) | 95.27 (3.37) | 96.21 (3.93) | <0.001 |
| Temperature, mean (SD), degrees F | 99.55 (1.43) | 99.78 (1.46) | 99.56 (1.43) | 99.20 (1.38) | <0.001 |

*Values are shown as SD (Standard Deviation)

ESR (Erythrocyte Sedimentation Rate)
